# Self-Assembled Multi-Epitope Peptide Amphiphiles Enhance the Immune Response against Enterovirus 71

**DOI:** 10.3390/nano10122342

**Published:** 2020-11-25

**Authors:** Yu-Gyeong Kim, Yunsu Lee, Joo Hee Kim, Sun-Young Chang, Jong-Wha Jung, Woo-Jae Chung, Hyo-Eon Jin

**Affiliations:** 1 College of Pharmacy, Ajou University, Suwon 16499, Korea; yoog607@ajou.ac.kr (Y.-G.K.); akbird1025@ajou.ac.kr (Y.L.); elisekim@ajou.ac.kr (J.H.K.); sychang@ajou.ac.kr (S.-Y.C.); 2 Research Institute of Pharmaceutical Sciences, College of Pharmacy, Kyungpook National University, Daegu 41566, Korea; jungj@knu.ac.kr; 3 Department of Integrative Biotechnology, Sungkyunkwan University, Suwon 16419, Korea; 4 Research Institute of Pharmaceutical Science and Technology, Ajou University, Suwon 16499, Korea

**Keywords:** peptide amphiphile, immune stimulator, vaccine, viral infectious disease, Enterovirus 71

## Abstract

Subunit vaccines consist of non-genetic material, such as peptides or proteins. They are considered safe because they have fewer side effects; however, they have low immunogenicity when used alone. We aimed to enhance the immune response of peptide-based vaccines by using self-assembled multimeric peptide amphiphiles (PAs). We designed two epitope PAs by conjugating epitope peptides from Enterovirus 71 (EV71) virus particle (VP) 1 and VP3 capsid proteins with different fatty acid chain lengths (VP1PA and VP3PA). These PAs self-assembled into supramolecular structures at a physiological pH, and the resulting structures were characterized using atomic force microscopy. Multi-epitope PAs (m-PAs) consisted of a 1:1 mixture of VP1PA and VP3PA solutions. To evaluate immunogenicity, m-PA constructs were injected with adjuvant subcutaneously into female Balb/c mice. Levels of antigen-specific immunoglobulin G (IgG) and IgG1 in m-PA-injected mice serum samples were analyzed using ELISA and Western blotting. Additionally, cytokine production stimulated by each antigen was measured in splenocytes cultured from immunized mice groups. We found that m-PA showed improved humoral and cellular immune responses compared to the control and peptide groups. The sera from m-PA immunized mice group could neutralize EV71 infection and protect host cells. Thus, self-assembled m-PAs can promote a protective immune response and can be developed as a potential platform technology to produce peptide vaccines against infectious viral diseases.

## 1. Introduction

Vaccines play an essential role in preventing infectious diseases. At present, 300 years after the first vaccine was released, vaccination prevents 2–3 million deaths annually, according to the WHO [1,2]. Vaccines aim to elicit a protective immune response in naïve hosts by exposing the host’s immune system to epitopes located on the pathogen before exposure to the infectious pathogen itself. The majority of vaccines against infectious diseases consist of inactivated or live attenuated pathogens [1,3]. These conventional vaccines are potent but are not always safe; they may cause the disease itself or contain contaminants that cause adverse effects [4,5]. However, subunit vaccines (vaccines based on peptide or protein subunits) containing only purified antigens or antigenic parts of the pathogen, and not the whole pathogen, have been developed. These are considered safer than conventional vaccines because of the lower risk of side effects [4,5,6]. However, the immunogenicity of subunit vaccines differs based on the antigen used and are generally less immunogenic than inactive or attenuated pathogens and often cannot confer optimal protection [6]. For this reason, various approaches to increase the immunogenicity of peptide vaccines have been tried. Peptides and small-molecule antigens presented on the surface of macromolecular assemblies, including epitopes presented as multimeric molecules, have emerged as a powerful strategy for eliciting immune responses with or without the use of adjuvants [7,8,9,10]. Antigenic materials composed of epitope peptides linked to lipopeptides, polymer-based nanoparticles, and virus-like particles have been found to induce robust antibody and cellular immune responses [7,8,9,10,11].

Self-assembling short peptides make useful synthetic soft materials for biomedical applications such as cell or drug delivery, regenerative medicine, and vaccine design [12,13,14]. Peptide amphiphiles (PAs) consist of peptides conjugated with spacer cross-linkers and hydrophobic long-chain fatty acids [12]. They can self-assemble in aqueous conditions into various supramolecular structures, such as nanotubes, micelles, nanovesicles, or cylindrical nanofibers [15]. The molecular size, shape, and charge are known to influence the immune response in peptide vaccines [14,16]. The advantage of self-assembled peptide materials is their significant multivalency [17], which enables the repetitive display of epitopes leading to enhanced antibody responses. This property is known to enhance the immunogenicity of fibrillized peptides [18,19], and peptide micelles [9].

Enterovirus 71 (EV71) is a single-stranded RNA virus of the enterovirus genus in the family *Picornaviridae* that causes hand, foot, and mouth disease. EV71 genome is about 7.4 kB and is encapsidated in an icosahedral protein capsid consisting of 60 copies of VP1, VP2, VP3, and VP4. EV71 is classified into A, B (B1–B5), C (C1–C5) subgenotypes, and subgenotype C4 is further subdivided into C4a and C4b [20]. VP1 is known to be the major antigenic target for subunit vaccines based on its antigenicity and antibody neutralizing capacity. The sequence YPTFGEHKQEKDLEY (residues 3059–3103, GenBank: EU703814.1) in VP1 is identical among all subtypes [21]. A new conformational neutralizing epitope of VP3, HYRAHARDGVFDYYT (residues 2237–2281, GenBank: EU703814.1), has been identified using in vitro and in vivo neutralization assays [22].

**Figure 1:**
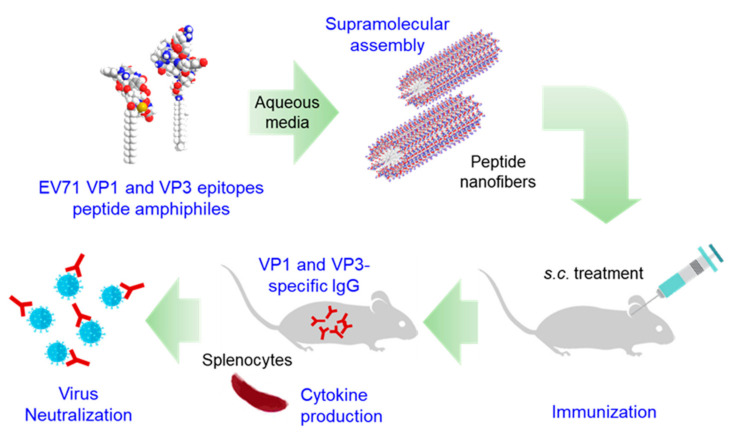
Experimental design to evaluate the self-assembly of Enterovirus 71 (EV71) virus particle 1 (VP1) and virus particle 3 (VP3) multi-epitope peptide amphiphiles and to analyze their immunogenicity in mice.

In this study, we proposed that PAs assembled into supramolecular structures as a multimeric strategy of epitope presentation could improve the immune responses. In our previous study, we designed PAs using VP1 epitope, which, when injected with alum into mice, showed a higher antibody response compared to peptides, but was still substantially low [18]. To overcome low efficacy, we chose two epitopes from EV7—virus particle 1 (VP1) and virus particle 3 (VP3)—and developed multi-epitope PA (m-PA)-based nanomaterials for each. Self-assembled VP1-epitope PA and VP3-epitope PA were mixed in equal amounts and developed as an immune modulator for the robust induction of immune responses to prevent EV71-induced infectious disease (i.e., hand, foot, and mouth disease) [23]. The structure of m-PAs against EV71 was imaged using atomic force microscopy (AFM). M-PA-induced humoral immune response was evaluated by measuring antigen-specific IgG and IgG1 and Western blotting, and the cellular immune response was evaluated by analyzing antigen-stimulated cytokine production in splenocytes culture from immunized mice. Furthermore, the neutralizing antibody titer of the m-PA-immunized mouse sera was assessed using in vitro virus neutralization assay in Vero cells (Figure 1).

## 2. Materials and Methods

### 2.1. Materials

EV71 C4a, the non-mouse adapted EV71 strain, Fuyang.Anhui.P.R.C/17.08/3 (GenBank: EU703814.1), was generously gifted by HK inno.N corp (Seoul, South Korea) for basic research [24]. Vero cell-line (Vero African green monkey kidney cells) was purchased from Korean Cell Line Bank (Seoul, South Korea). AddaVax^®^ was purchased from Invivogen (San Diego, CA, USA). Kanamycin was purchased from Sigma-Aldrich (St. Louis, MO, USA). Luria-Bertani (LB) broth, Bacto^®^ Agar (BD Difco, NJ, USA), and isopropyl-β-D-1-thiogalactopyranoside (IPTG) were purchased from Bioneer (Daejeon, South Korea). D-Plus™ Protein Gel Staining Solution was purchased from Dongin Biotech Co. (Seoul, South Korea). The Polyvinylidene fluoride (PVDF) membrane, ammonium persulfate, 10% sodium dodecyl sulfate (SDS) solution, and 30% acrylamide/bis solution were purchased from Bio-Rad (Hercules, CA, USA). Horseradish peroxidase (HRP)-conjugated rabbit anti-mouse IgG and IgG1 antibodies were purchased from Abcam (Cambridge, MA, USA.) and Southern Biotech (Birmingham, AL, USA), respectively. Pierce TMB substrate kits, HIS-bind^®^ purification resin, and sulforhodamine B (SRB) were purchased from Thermo Fisher Scientific (Waltham, MA, USA).

### 2.2. Methods

#### 2.2.1. Design and Synthesis of Virus Epitope-PAs

The epitope sequences YPTFGEHKQEKDLEY (residues 3059–3103) and HYRAHARDGVFDYYT (residues 2237–2281) were selected from EV71 capsid proteins 1 and 3, respectively (GenBank: EU703814.1) [22]. VP1 and VP3 epitope PAs (VP1PA and VP3PA) were synthesized using standard Fmoc chemistry-based solid-phase peptide synthesis using *N*-Fmoc-amino acids and Rink resins [12]. The VP1 epitope peptides and spacers (GGGCC) were conjugated to palmitic acid (C16), and the VP3 epitope peptides and spacers (GGGCC) were conjugated to stearic acid (C18), via their C-terminal lysine. The control PAs (CPAs) were constructed with cross-linkable spacers (GGGCCK) conjugated to long-chain fatty acids (palmitic acid or stearic acid) without the epitope peptide [12]. PAs and CPAs were synthesized by Peptron (Daejeon, South Korea) and Biostem (Ansan, South Korea), respectively [25].

#### 2.2.2. Self-Assembly of PAs for Supramolecular Structures

The self-assembled PAs were prepared by pH control as described earlier [12,18]. VP1PA, VP3PA, CPA-16, and CPA-18 solutions were prepared at concentrations of 6 mg/mL in deionized water, and the pH values of the solutions were adjusted to 10 for solubilization. The solutions were then mixed to make VP1PA:CPA-16 and VP3PA:CPA-18 at a molar ratio of 6:4 each. To trigger the self-assembly of PA, the pH values of the solutions were gradually decreased to 7.4 using 0.01 N HCl.

#### 2.2.3. Supramolecular Structures of PAs Analyzed Using AFM

Self-assembled VP1PA and VP3PA solutions were applied on a silicon wafer and dried at room temperature (RT). AFM images were collected in the non-contact mode using an XE7 AFM (Park Systems, Suwon, South Korea) and analyzed using the XEI 4.3.4 software (Park Systems).

#### 2.2.4. Purification of EV71 Capsid Proteins

EV71 recombinant capsid protein 1 (rVP1) was cloned in pBHA vector using a synthetic method by Bioneer (Daejeon, South Korea) and sub-cloned to pET28b vector in our lab. The DNA sequence for the recombinant capsid protein 3 (rVP3) was synthesized and cloned in pET28b by Bionics (Seoul, South Korea). Cloned plasmids were transformed into *Escherichia coli* BL21-CodonPlus (DE3)-RIL competent cells (Agilent Technologies, U.S.A.) for protein expression. rVP1 and rVP3 were expressed in LB medium containing kanamycin (50 μg/mL) at 37 °C and 220 rpm. When the cultured cells reached an optical density (OD, 600 nm) of 0.5, IPTG was added to the culture media (250 μM for rVP1 and 500 μM for rVP3) and the cells were incubated for another 4 h. The cells were harvested and lysed in lysis buffer containing 20 mM Tris HCl (pH 8.0), 100 mM NaCl, and cOmplete™ mini EDTA-free protease inhibitor cocktail (Sigma-Aldrich, MO, USA) and disrupted by ultrasonication (Branson Sonifier cell disruptor, Hielscher, Sonics & Materials, Inc., Newtown, CT, USA) on ice. After centrifugation, rVP1 and rVP3 were purified using an HIS-bind^®^ resin (Thermo, USA) according to the manufacturer’s instructions, and were subsequently dialyzed with phosphate-buffered saline (PBS) [18,24]. The size of rVP1 and rVP3 was confirmed via SDS-polyacrylamide gel electrophoresis (PAGE).

#### 2.2.5. Immunization

Six-week-old female Balb/c mice (Orient Bio, Sungnam, South Korea) were selected and allocated into experimental groups (four mice per group). To induce an immune response, 100 μg of each antigen (m-peptide, a 1:1 mixture of VP1 and VP3 epitope peptides; m-PA, a 1:1 mixture of VP1 and VP3 epitope PAs; m-protein, a 1:1 mixture of rVP1 and rVP3 proteins) in 50 μL phosphate-buffered saline (PBS) along with 50 μL of adjuvant (AddaVax^®^) was subcutaneously injected into mice in the first week. PBS was injected into the mice in the negative control group [8]. A further 50 μg of each antigen was injected into the mice at weeks 3 and 5 for boosting. Blood samples were collected from the abdominal artery of immunized mice 7 days after the last injection. The serum was separated by centrifugation and stored at −20 °C until use. After blood sample collection, the spleens were isolated for measuring the spleen size and for the cytokine assays. All animal experiments were approved by the Institutional Animal Care and Use Committee of Ajou University (IACUC).

#### 2.2.6. Enzyme-Linked Immunosorbent Assay (ELISA)

EV71 rVP1 and rVP3-specific immune responses were evaluated by ELISA. A 96-well plate was coated with 2 μg/well of rVP1 or rVP3 protein in 50 mM carbonate-bicarbonate buffer (pH 9.6) and incubated overnight at 4 °C with gentle rocking. The wells were treated with blocking buffer consisting of 1% bovine serum albumin (BSA) in 0.1% Tween 20 in PBS (0.1% PBS-T) for 1 h at RT. Serum samples were serially diluted in 0.1% PBS-T containing 0.1% BSA and incubated in the wells for 1 h at RT. The plate was then washed with 0.3% PBS-T and incubated with HRP-conjugated rabbit anti-mouse IgG or IgG1 antibody (1:5000) for 1 h at RT. After washing, 3,3’,5,5’-tetramethylbenzidine (TMB) solution (Pierce, MA, USA) was added, followed by the addition of 2M sulfuric acid stop-solution. Absorbance was measured at 450 nm using an ELISA reader (Synergy H1, BioTek, VT, USA) [26].

#### 2.2.7. Western Blot Analysis

The humoral immune response to antigen injection was assessed by western blotting. Excel Band™ Enhanced 3-color high range protein markers (SMOBiO, Hsinchu City, Taiwan) and 2 μg of purified rVP1 or rVP3 proteins were loaded into each well of a 12% SDS-polyacrylamide gel. The proteins were separated by electrophoresis, electroblotted onto PVDF membranes (Bio-Rad, CA, USA), and blocked with 5% skimmed milk in 0.1% PBS-T overnight at 4 °C [27]. Serum samples were diluted 1:100 with 1% skimmed milk in PBS-T and added to the membranes, and incubated for 1 h at RT. The membranes were then treated with rabbit anti-mouse IgG antibodies conjugated with HRP (1:5000, Abcam) for 1 h. IgG signals were detected using a 3-amino-9-ethylcarbazole solution (Alfa Aesar, MA, USA).

#### 2.2.8. Cytokine Assay

Single-cell suspensions of splenocytes were prepared from the spleens of immunized mice using a 70-μm strainer, and cultured with RPMI 1640 medium (Welgene, Daegu, South Korea). Isolated splenocytes were resuspended in RPMI 1640 containing 10% fetal bovine serum, 100 U/mL penicillin, and 100 μg/mL streptomycin (Welgene, Daegu, South Korea) and plated at 2 × 10^6^ cells per well in 96-well round-bottom culture plates (Corning, NY, USA). Cells were cultured for 2 days with 50 μg of each antigen in 150 μL of medium. After 48 h of incubation, the supernatants of the treated cultures were collected and frozen at −20 °C until assayed. The levels of interleukins (IL) (IL-2, IL-10, IL-17A) and interferon-gamma (IFN-γ) were quantified using an MILLIPLEX MAP Immunology Multiplex Assay^®^ (Sigma-Aldrich, St. Louis, MO, USA) by Komabiotech (Seoul, South Korea) [8].

#### 2.2.9. Neutralization Assay

Sera were two-fold serially diluted from 1:10 to 1:320 and mixed with TCID_50_ (median tissue culture infective dose) of EV71 for 1 h at 37 °C. Then, solutions were added to Vero cells (2 × 10^4^ cells/well) in 96-well plates and incubated at 37 °C with 5% CO_2_. After 72 h, the cytopathic effect (CPE) of EV71 on Vero cells was observed using a digital light microscope DS-Fi2 (Nikon, Tokyo, Japan). The cell viability was measured by sulforhodamine B (SRB) assay using CPE reduction [28]. Serum dilutions were tested in triplicates and the neutralization titers were calculated as the highest dilution that protected the cells from CPE.

#### 2.2.10. Statistical Analysis

All data are expressed as mean ± standard error (SEM). Statistical analysis was performed using one-way or two-way analysis of variance (ANOVA) along with Tukey’s multiple comparisons test, conducted using GraphPad Prism 7 (GraphPad Software, La Jolla, CA, USA). A *p*-value less than 0.05 was considered to be statistically significant.

## 3. Results and Discussion

### 3.1. Design and Self-Assembly Properties of Epitope-PAs

**Figure 2:**
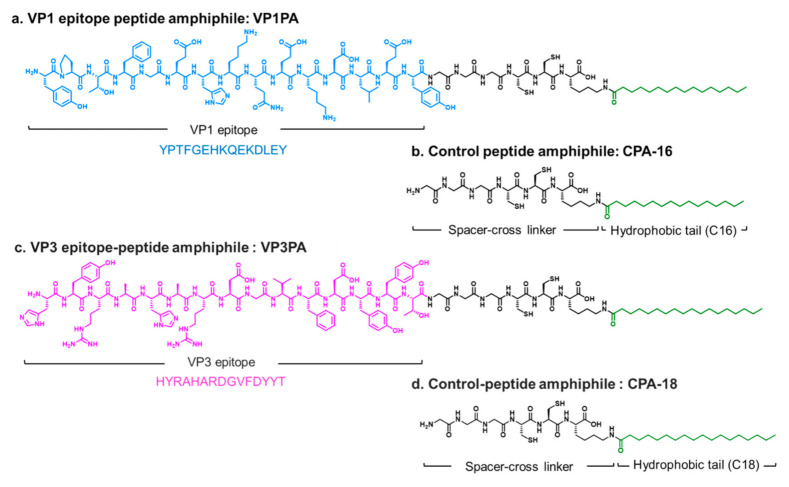
Structures of EV71 epitope peptide amphiphiles and control peptide amphiphiles. (**a**) EV71 VP1 epitope peptide amphiphile (VP1PA) and (**b**) control peptide amphiphile (CPA-16). (**c**) EV71 VP3 epitope peptide amphiphile (VP3PA), and (**d**) control peptide amphiphile (CPA-18).

**Figure 3:**
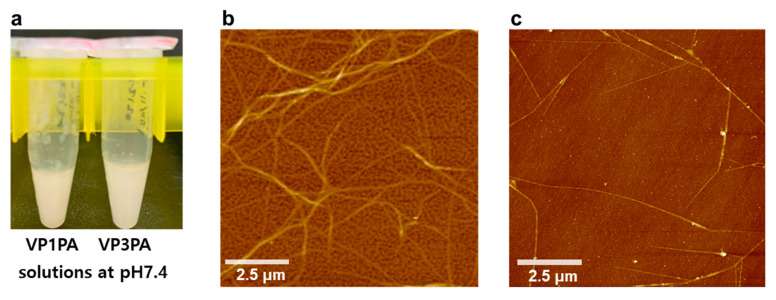
Characteristics of self-assembled epitope peptide amphiphiles. (**a**) Images of mixed solutions. 6 mg/mL in deionized water at the molar ratio of VP1PA:CPA-16 = 6:4 (VP1PA) and VP3PA:CPA-18 = 6:4 (VP3PA) at pH 7.4. Mixed solution of VP1PA:CPA-16 (**b**) and VP3PA:CPA-18 (**c**) forms nanofibres at pH 7.4 (1 μg/mL) imaged by atomic force microscopy (AFM).

We selected two neutralizing epitopes from EV71, VP1 and VP3, for PA-based vaccine design. Two types of epitope-PAs were designed: YPTFGEHKQEKDLEY-GGGCCK-palmitic acid (VP1PA) and HYRAHARDGVFDYYT-GGGCCK-stearic acid (VP3PA) (Figure 2). VP1PA consists of VP1 epitope and a cross-linkable spacer (GGGCCK) conjugated to palmitic acid (C16) (Figure 2a) [12,18]. VP3PA consists of VP3 epitope and a cross-linkable spacer (GGGCCK) conjugated to stearic acid (C18) (Figure 2c). The CPAs were GGGCCK-palmitic acid (CPA-C16) and GGGCCK-stearic acid (CPA-C18) without the epitope peptides (Figure 2b,d). Remarkably, both the solution of VP1PA:CPA-16 and VP3PA:CPA-18, each at a molar ratio of 6:4, turned turbid, and a white precipitate was observed at the physiological pH 7.4 (Figure 3a). AFM images showed that VP1PA and VP3PA assembled into nanofibers with relatively uniform diameter (131.8 ± 16.8 nm for VP1PA and 77.1 ± 17.8 nm for VP3PA) at a concentration of 1 μg/mL in drop-cast samples (Figure 3b,c). AFM images revealed that the assembled epitope PAs retained nanofibrous morphology with a relatively uniform diameter, implying that the epitope peptide-based assembled structures exhibit size stability without any problematic aggregation.

PAs are surfactant molecules that assemble into supramolecular structures, such as spherical micelles, nanofibers, vesicles, and inverted micelles in aqueous conditions because of the difference in the critical packing parameter [15]. The molecular packing parameter is widely used to understand and predict molecular self-assembly in surfactant solutions. The molecular packing parameter is defined as v_o_/a_e_l_o_, where v_o_ is the tail volume, l_o_ is the tail length, and a_e_ is the head equilibrium area per molecule at the aggregate surface. The concept of the molecular packing parameter emphasizes the importance of the surfactant headgroup in predicting the size and shape of equilibrium aggregates of surfactants. In addition, R. Nagarajan reported that the surfactant tail also controls the equilibrium aggregate structures [29]. In this study, we designed two nanofibers for peptide-based vaccine. We observed that the VP1 epitope linked to palmitic acid (C16) and the VP3 epitope linked to stearic acid (C18), showing assembly of nanofibers. VP3 epitope linked to palmitic acid (C16) was not clearly assembled into nanofibers (data not shown). This may be due to the difference in head equilibrium area per molecule (a_e_) at the aggregate surface of VP1 and VP3 epitopes.

### 3.2. Spleen Morphology by the Immunization with EV71 Epitope m-PA

**Figure 4:**
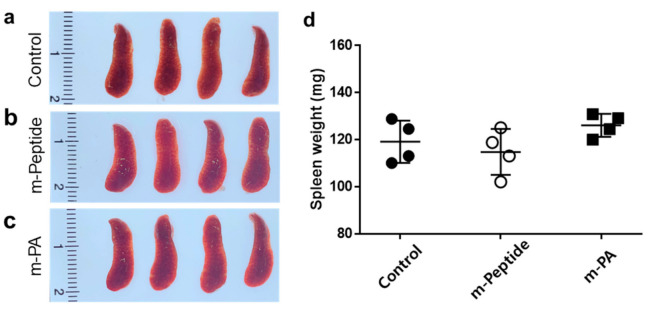
Comparative spleen size and morphology. (**a**–**c**) Appearance and relative size of the spleens isolated from the mouse groups. (**d**) Relative differences in the spleen weights among the mouse groups. Control, phosphate-buffered saline (PBS) injected; m-peptide, VP1 and VP3 multi-epitope peptide; m-PA, VP1 and VP3 multi-epitope peptide amphiphiles.

Balb/c mice were immunized with antigens (m-peptide, and m-PA) three times every other week, and the sera and spleens of the immunized mice were collected seven days after the last injection. Next, we evaluated whether vaccination with epitope PA affected mice spleen. There was no significant difference in the spleen weight between the control and the antigen-immunized mice groups (Figure 4).

### 3.3. Immunization with EV71 Epitope m-PA Elicits Anti-EV71 Antibody Production

**Figure 5:**
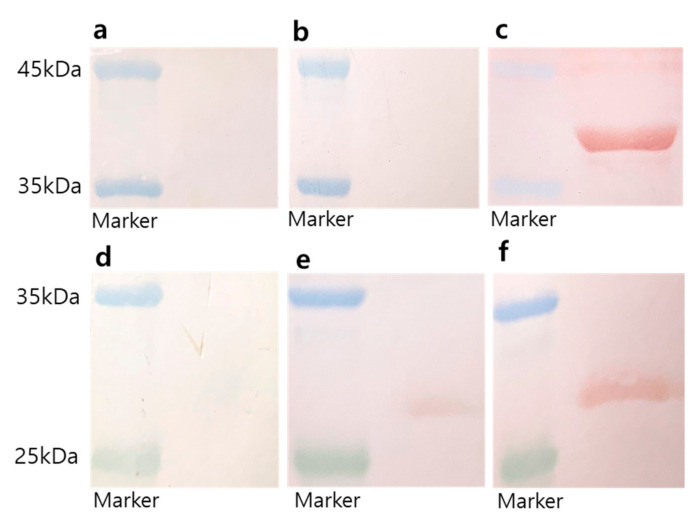
Western blot analysis of serum samples from each group of immunized mice. (**a**–**c**) rVP1 (approximately 37 kDa) and (**d**–**f**) rVP3 protein (approximately 26 kDa)-blotted membranes treated with the diluted sera (1:100) obtained from mice immunized with (**a**,**d**) PBS (control), (**b**,**e**) m-peptide, and (**c**,**f**) m-PA. Red bands indicate rVP1 or rVP3 proteins that reacted with the serum. Blue bands are markers for 45 and 35 kDa, while green bands are the marker for 25 kDa.

Immune effector functions induced by vaccines are essentially antibodies produced by B cells that can specifically bind to pathogens or toxins [30]. We purified EV71 rVP1 and rVP3 from *E. coli* and used them to measure EV71 virus particle-specific antibodies. IgG antibody against rVP1 and rVP3 could neutralize EV71 and may prevent EV71 infection. To analyze the presence of such antibodies, the rVP1 and rVP3 proteins were blotted on the membranes, and the antibody response against these proteins was determined from the sera of immunized mice by western blotting (Figure 5). The rVP1 and rVP3 proteins migrate around 37 kDa and 26 kDa, respectively, as determined using SDS-PAGE (data not shown). The sera from the control PBS-injected mice did not exhibit rVP1- or rVP3-specific IgG, and thus, did not show a red band on the blotted membranes (Figure 5a or Figure 5d, respectively). In contrast, the sera from the m-PA-immunized mice showed red bands on the rVP1 and rVP3 blotted membrane (Figure 5c,f). The sera from the m-peptide group showed no band or a faint band on rVP1 and rVP3 blotted membrane, while the m-PA groups displayed a thick band (Figure 5b,e). These results support the suggestion that m-PA can induce a humoral immune response, thereby enhancing the overall immune response, while the peptides themselves cannot (Figure 5).

### 3.4. m-PA Conjugation Improved Humoral Immune Response to EV71 Epitopes

**Figure 6:**
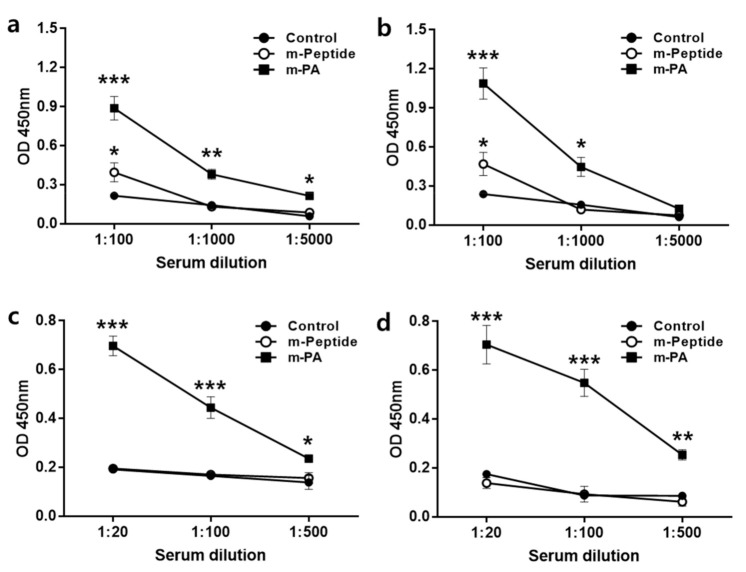
Humoral immune response induced by EV71 antigens (m-peptide, m-PA). The level of (**a**) rVP1, and (**b**) rVP3-specific immunoglobulin G (IgG) production was detected by using indirect enzyme-linked immunosorbent assay (ELISA). The level of (**c**) rVP1, and (**d**) rVP3-specific IgG1 was measured by ELISA. All data are presented as mean ± SEM. Significant difference from the control group are indicated by * *p* < 0.05, ** *p* < 0.01, *** *p* < 0.001 (*n* = 4).

The levels of antigen-specific IgG and IgG1 (a major IgG subclass that neutralizes EV71 [31]) antibodies were measured from the sera of the three animal groups by ELISA (Figure 6). Sera from m-PA-immunized mice were obtained and analyzed to measure rVP1- and rVP3-specific IgG. The m-PA group showed significantly higher levels of rVP1-specific IgG at all dilutions than the control group (Figure 6a), while the m-peptide group only showed a significant difference from the control group at 1:100 diluted sera (Figure 6a). Additionally, the m-PA group showed significantly higher levels of rVP3-specific IgG than the control group at 1:100 and 1:1000 diluted sera (Figure 6b). In contrast, the m-peptide group showed a significant difference from the control group only with 1:100 diluted serum (Figure 6b). Furthermore, the m-PA group had significantly higher rVP1 and rVP3-specific IgG1 levels than the control group, while the m-peptide group showed no differences (Figure 6c,d). These results indicate that the nanofiber forms of m-PA with multimeric epitope approach improve immunogenicity compared to the peptide alone.

### 3.5. EV71 Epitope m-PA Induces a Cell-Mediated Immune Response

We evaluated the ability of m-PAs to activate the cell-mediated immunity by analyzing the production of cytokines in splenocytes. Splenocytes were harvested from immunized mice seven days after the last injection and cultured for 48 h in the presence of each antigen (m-peptide and m-PA). The levels of IFN-γ, IL-2, IL-10, and IL-17A were determined in the supernatants of the splenocyte culture medium using the MILLIPLEX MAP bead-based multiplex assay (Table 1). The m-PA group showed significantly higher IFN-γ and IL-2 production from splenocytes compared to control and m-peptide groups. Moreover, the m-PA group showed increased IL-10 and IL-17 production from splenocytes compared to control and m-peptide groups, but this difference was not statistically significant. These results indicate that m-PA, unlike m-peptide, can induce a cell-mediated immune response.

The immune response to subunit vaccines depends on the antigen used. Protein antigens typically trigger T-cell-dependent adaptive immune responses [32]. Although peptide antigens alone induce a low level of immune response, our multimeric approach using m-PA could induce cell-mediated immunity (Table 1). To overcome insufficient immunostimulatory capabilities, an effective adjuvant can be used with m-PA. Alum (aluminum salts) is the most commonly used adjuvant and has been the only adjuvant used in human vaccines for almost 100 years [33]. Alum stimulates a strong Th2 response via a depot effect and the activation of antigen presenting cells, but it is rather ineffective against pathogens that require Th1-mediated immunity. In this study, we used AddaVax^®^ as an adjuvant. AddaVax^®^ has components similar to the newly licensed adjuvant, MF59^®^, which is a squalene-based oil-in-water emulsion and a stimulator of both Th1 (cellular) and Th2 (humoral) immune responses [34,35]. Likewise, m-PA might be used with other potent stimulators of Th1 and Th2 immune responses to achieve sufficient immune induction. Furthermore, the choice of adjuvants in peptide vaccination is critical because epitopes designed with specific conformations may require adjuvants that do not denature or emulsify the antigens [32]. Predicting which adjuvant will elicit the best immune response against an antigen is difficult, therefore screening for the best-suited adjuvant is helpful. Thus, with m-PA, it is important to use an adjuvant that maintains the assembled presentation of the multimeric epitopes while improving m-PA’s immune stimulatory effect.

### 3.6. EV71 Epitope m-PA-Treated Sera Protects Cells from EV71 Infection

**Figure 7:**
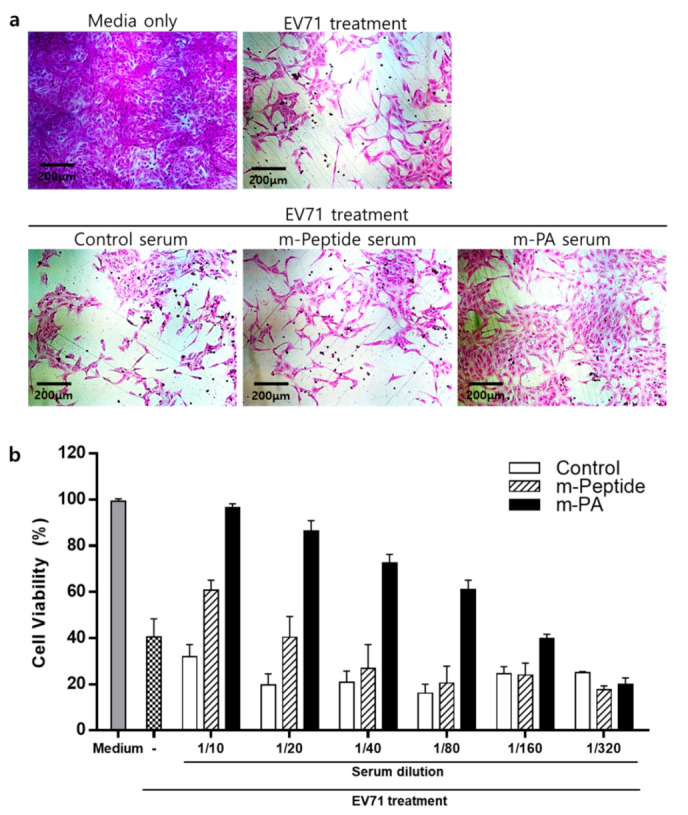
Serum neutralization assay. VP1 and VP3-specific antibodies can neutralize EV71 and protect Vero cells. EV71 neutralizing effect in sera was assessed using cytopathic effect assay (using SRB). (**a**) Representative images of Vero cells treated with TCID_50_ of EV71 in 80-fold diluted control or m-PA serum. (**b**) Cell viability was assessed after infection with EV71 and the two-fold serially diluted serum. The highest dilutions in which more than 50% of cells survived after infection with TCID_50_ of EV71 were considered as the neutralization titers.

Next, we assessed whether sera containing rVP1 and rVP3-specific antibodies can neutralize EV71 using a modified cytopathogenic effect assay (SRB assay). Enterovirus 71 infection induces progressive cellular morphological changes and apoptosis in Vero cells [36]. The effect of sera from control, m-PA, and m-peptide groups on EV71-induced CPE was observed in Vero cells using a microscope (Figure 7a). Cells that were not infected with EV71 were treated with media (negative control). Serum from m-PA treated group showed more neutralization activity against EV71 infection compared to control and m-peptide treated groups in Vero cells (Figure 7a,b). Neutralizing antibody titers for m-peptide serum was 1:10 and m-PA serum was 1:80 (Figure 7b). As shown in Figure 7b, control serum did not show any neutralization effect even at 1:10 dilution (the lowest dilution tested). Collectively, these results clearly suggest that immunization of mice with m-PA and adjuvant successfully produced protective neutralizing antibodies against EV71 infection.

In this study, we designed an m-PA-based subunit vaccine using two neutralizing epitopes from EV71, VP1 and VP3. The epitope sequence YPTFGEHKQEKDLEY from VP1 is identical among all EV71 subgenotypes (including A, B (B1–B5), and C (C1–C5)) [21]. Therefore, this m-PA-based vaccine design might be used to protect against all subtypes of EV71 in viral infectious disease, including hand, foot, and mouth disease.

EV71 C4a genotype was used in this study for the verification of m-PA effect because EV71 C4a is prevalent in Northeast Asian countries including China and South Korea. EV71 C4a, unlike other EV71 subgenotypes, cannot infect both neonate and adult ages of wild-type mice. Neonate transgenic mouse expressing human scavenger receptor B2 (hSCARB2), a viral receptor for EV71, was developed [37,38]. However, neonatal mice are too young for us to be able to investigate the protective immunity of vaccine immunization against viral challenge, and hence a passive transfer model using neonate Tg mice needs to be applied. A 3-week-old Mongolian gerbil (*Meriones unguiculatus*) has been used as a model for studying EV71 C4a infection [39,40]. In this study, we verified our proof of concept using serum neutralization assay to determine whether enhanced production of neutralizing antibodies induced by PA-based vaccine design could efficiently reduce viral infection in the host cells. Serum neutralizing titer to EV71 C4a showed high correlation in an in vivo protection assay [24], implying that m-PA may protect host cells in vivo. Protective effects of both antibodies and T cells induced by m-PA will be further investigated in the Mongolian gerbil system.

## 4. Conclusions

Peptide vaccines are a safer and more economical alternative to traditional vaccines, which are usually composed of dead or attenuated pathogens, inactivated toxins, and recombinant subunits that may contain endotoxins or contaminants from culture and processing steps. However, despite their advantages, peptides have low immunogenicity when used alone or even with adjuvants. To enhance the immunogenicity of peptide-based vaccines, we designed multimeric, m-PA-based vaccines by conjugating neutralizing epitope peptides (VP1 and VP3) from EV71 capsid proteins with different fatty acid chain lengths. At a physiological pH, m-PA constructs self-assembled into nanofibers. The m-PA vaccinated groups demonstrated improved humoral immune response and exhibited higher rVP1- and rVP3-specific IgG and IgG1, compared to the peptide-only-treated groups. Furthermore, IFN-γ and IL-2 production were significantly increased by m-PA stimulation in splenocytes, implying that m-PAs could also induce cellular immune response. Thus, the self-assembled m-PAs we developed promoted a protective immune response against viral infectious diseases and can be developed as a platform technology to produce vaccines. Further research is required to amplify the immunostimulatory capabilities using appropriate adjuvants. Moreover, the m-PA-based vaccine can be further developed for clinical use in immunization against hand, foot, and mouth disease.

## Figures and Tables

**Table 1:** Cytokine levels in the splenocyte cultures obtained from immunized mice upon antigen stimulation.

Cytokine (pg/mL)	Control	m-Peptide	m-PA
INF-γ	29.54 ± 10.38	29.74 ± 19.87	1353.9 ± 463.9 *^,^^†^
IL-2	19.97 ± 4.39	18.93 ± 3.46	59.29 ± 11.63 **^,^^††^
IL-10	5.94 ± 0.79	7.49 ± 1.67	198.6 ± 119.5
IL-17A	2.07 ± 0.93	3.75 ± 1.30	74.75 ± 25.51

Cytokine quantification. Levels of splenocyte-secreted cytokines upon stimulation with each antigen were determined using bead-based multiplex assays. All data are presented as mean ± SEM (*n* = 4). Significance is indicated as follows: * *p* < 0.05, ** *p* < 0.01 compared to control group, ^†^ *p* < 0.05, ^††^ *p* < 0.01 compared to the m-peptide group.
